# Proceedings of the Massachuseetts Medical Society

**Published:** 1860-07

**Authors:** 


### PROCEEDINGS OF SOCIETIES.

### ARTICLE XXVII.

*Proceedings of the Massachuseetts Medical Society.*

THE Annual Meeting of the Massachusetts Medical Society was held in the hall of the Lowell Institute, Boston, on Wednesday, May 30th, 1860, at 10, A. M. The President, Dr. John Homans, in the chair.

After the usual business, Dr. C. C. Holmes, of Milton, offered the following resolution, which was unanimously adopted :—*Resolved,* That a committee of five be appointed to propose what action, if any, is proper, on the part of this Society, respecting the disease now prevailing among the cattle of this commonwealth, and that the committee be instructed to report at the earliest moment practicable. The Chair nominated Drs. H. I. Bowditch, J. Sargent of Worcester, J. G. Metcalf of Mendon, S. Whitney of Framingham, and C. C. Holmes of Milton.

Three communications were then presented :—one upon Neuroma, by Dr. Alfred Hitchcock, of Fitchburg ; another upon the Zymoses of 1859, by Dr. Ephraim Cutter, of Woburn ; and a third upon the subject of Vaccination under the direction of the Middlesex East District Medical Society, by Dr. A. Chapin, of Winchester.

In reference to a memorial presented to the Councillors at the stated meeting in February, 1860, the following resolution was unanimously adopted :—*Resolved,* That the Society assume the payment of the expenses of the several suits brought by Ira Barrows against Drs. B. Carpenter, L. V. Bell and D. H. Storer, and hold the memorialists harmless from all pecuniary liabilities growing out of them.

The committee appointed to consider the subject of the disease now prevailing among cattle, reported the following preamble and resolution, which were unanimously adopted ; Whereas a disease, the nature of which is not well understood, and the treatment of which has been to a remarkable degree unsuccessful, is now prevailing among cattle in this commonwealth, and whereas the Legislature is this day convened to consider this specific object, therefore, *Resolved,* That a committee of nine fron the Mass. Med. Society be appointed by the Chair to urge upon the Legislature the establishment of a scientific commission to investigate said disease. The Chair appointed Drs. J. Bigelow, Geo. Hayward, H. I. Bowditch, and J. B. S.

Jackson, of Boston; O. Martin, of Worcester; J. C. Bartlett, of Chelmsford; J. Gardner, of Pawtucket; C. P. Fiske, of Sturbridge; and J. G. Metcalf, of Mendon.

The Chair nominated the following Committee on Scientific Communications for the ensuing year:—Drs. John G. Metcalf, of Mendon, H. I. Bowditch of Boston, and George Choate of Salem, and they were appointed.

The appointed hour having arrived, Dr. O. W. Holmes delivered the Annual Address. This was upon a subject which has always especially occupied the most active minds of the profession in this city—the use and abuse of drugs. As it is soon to be published, we will not now attempt an analysis of it.

After a vote of thanks to Dr. Holmes for his eloquent and interesting discourse, the Society assembled in Faneuil Hall, where the usual dinner was served. A prayer was offered by the Chaplain, Rev. Rufus Ellis.

On the removal of the cloth, the members were welcomed by the Anniversary Chairman, Dr. D. H. Storer, as follows:—

Welcome, Fellows of the Massachusetts Medical Society. Welcome to this annual festival. Let us forget, for the passing hour, our professional responsibilities, and devote it to social intercourse. Let our meeting be what a meeting of old and tried friends should be, replete with gratitude and hope; *gratitude*, that our beloved Institution should have existed for so long a period, constantly increasing in strength and usefulness; *hope*, that its future career may be more brilliant than its past.

With the history of our Society you must all be familiar—with its early struggles, its gradual growth, its final success. At no previous period have its members been more numerous; never has there existed a more honorable intercourse between them than at the present time. Since our last annual meeting, no pestilence has swept through our ranks; but few are the links which are missed from the chain.

We see the same cheerful contenances—we feel the same cordial grasp, which have heretofore greeted us—and, thank Heaven, he is still spared to us, an exemplar, and councillor and friend, whom we all honor, whom we all love ! Joyous, indeed, then, must be our Thanksgiving day ! most hearty must be our mutual congratulations !

I would implore *Our Society's continued prosperity*—to attain which no one will feel a greater desire—no one can make a greater effort, than our most faithful, excellent President.

After a few appropriate words from Dr. Homans, the Chairman offered the following :

*The Orator of the Day*—never disappointing, in any intellectual effort, the most sanguine expectations—our community's pride, his friends' idol.

Dr. Holmes responded in his usual happy manner.

The Chairman then said, in the discharge of our every-day professional duties we are constantly needing the services of, we are constantly receiving invaluable aid from our brethren of the other learned professions. A distinguished representative of each of these professions is with us to day. They are each too well-known to require any formal introduction. I will therefore merely recall to your minds and your hearts

*The liberal, faithful Christian gentlemen* who aid us so much in our efforts to relieve suffering humanity—and who, when our efforts are unavailing, strive, by virtue of their office, to prepare the weary spirit for its final home.

This called out the Rev, Mr. Ellis, who alluded to the annual address in a complimentary manner, but remarked that as he listened, the thought occurred to him that the words of the speaker might produce upon some physicians an effect similar to that produced upon clergymen by the advancement of very liberal religious views. He did not believe however, that the doctor was in any real danger from his brother physicians, but would recommend the appointment of a committee whose duty it should be to protect him from the apothecaries. He fully recognized the relation existing between physicians and clergymen, and very aptly remarked that the encouragement of quackery by some of his clerical brethren could only be compared to the action of the deadheads upon a railway train, who showed their appreciation of favors by recommending a rival line.

The following sentiment was then offered :—

*The Sciences of Medicine and Law*—often indispensable together, to established rights which have been alienated, to remove unjust suspicion, to confirm an injured one's innocence or a depraved one's infamy, to determine at what time and by what means a life has been destroyed—to elucidate, in word, what is involved in mystery.

It would have afforded us much pleasure, gentlemen, to have introduced to you upon this occasion, the eminent legal adviser, who, during the entire last decade of your history, has held himself ready at all times to protect your interests and defend your honor ; but paramount duties have controlled him, and he remains at his post in the Capitol of our Republic, one of the ablest advocates of the rights of man—I refer to that accomplished lawyer and honest man, Thomas D. Elliott, of New Bedford. We are honored to-day by the presence of Chief Justice Shaw, of whom it has been well said by a distinguished member of the bar, "the high station which he occupies has been filled by him as none other has filled it, as none other in our day can."

This was responded to by Chief Justice Shaw, who acknowledged in a most feeling manner his cordial reception, and paid a warm tribute to the medical men whose testimony is so often essential in courts of justice.

The following was responded to by Dr. Fiske, of Fiskdale.

*The sagacious, judicious, upright country practitioner*—the most welcome guest in every family within his circuit: The most valuable, the most valued member of the community in which he lives.

He spoke as representing country practitioners, and showed, in his own person, that they are in every way able to speak for themselves.

The following called out Dr. Jacob Bigelow.

*The rational physician*—who knows that all real science is based upon the unvarying laws of Nature—that if Truth is sought for, her dictates alone must be followed.

We regret very much that we cannot give the remarks of those gentlemen who responded to the following sentiments from the Chairman.

*The genial, sympathizing, warm-hearted Doctor*—whose very presence in the sick chamber is sunshine, and whose kind and faithful attentions are the most effectual balm.

Responded to by Dr. Reynolds.

Great as has been the light shed upon the subject of medical jurisprudence during the last quarter of a century, the student proudly claims for America, its most vivifying Ray.

Dr. Ray being absent, answered through Dr. U. Parsons, of Providence, R. I.

We see before us to-day, one who has been in labor for more than fifty years ; protracted, tedious, however may have been his travail, he exhibits no symptoms of *nervous exhaustion*, he presents no appearance of *puerperal mania*, but displays the same vivacity and other peculiarities which characterized him when we listened to his instructions thirty-five years ago. We rejoice to see him here, to know that he still labors, well.

Dr. Channing replied.

Our brethern throughout the length and breadth of the land, who are devoting their lives to the amelioration of the condition of the insane. Holy is their mission. Most nobly is it being performed. Let us, as one man, God speed them in their work.

This was responded to by Dr. Walker of South Boston.

*Professional integrity*—never more strikingly illustrated than by his career, which prompted one of our keenest members, when advisng a young man just entering upon the duties of life, to concentrate all he considered necessary to say, in the single word be *Ware*.

Dr. Ware being absent, this was replied to by Dr. Gardner of Pawtucket.

We have just learned that a gentleman is with us, sent as a delegate to our meeting by the Connecticut Medical Society. In your name, I would offer him a cordial welcome.

To which happy sentiment Dr. Noyes responded.

---

Dr. Holmes' Address.—As the Address deliverd by Dr. Holmes last week, has been variously and erroneously reported in some of the public papers, the attention of our readers is directed to the following note on the subject from Dr. H.:

Messrs. Editors,—I beg leave to say that I prepared an abstract of my address before the Mass. Medical Society for the Boston [daily] Journal, and disclaim all responsibility for opinions attributed to me in any other report of the Address. Yours, very truly,

O. W. Holmes.

*June 4th*, 1860.

